# UK Optometrists’ Professional Learning Needs Toward Engaging with Myopia Control Interventions

**DOI:** 10.22599/bioj.341

**Published:** 2024-02-07

**Authors:** Wan Elhami Wan Omar, Fiona Cruickshank, Hema Radhakrishnan

**Affiliations:** 1 Faculty of Biology, Medicine and Health, The University of Manchester, United Kingdom; 2 Faculty of Health Sciences, Centre For Optometry Studies, Universiti Teknologi MARA, (UiTM) Selangor, Malaysia; 3 Faculty of Biology, Medicine and Health, The University of Manchester, GB

**Keywords:** optometrists’ skills, learning needs, myopia control, myopia management

## Abstract

**Purpose:**

This study aimed to explore the support that UK optometrists feel they require to facilitate their engagement with myopia control intervention.

**Methods:**

A self-administered online survey was distributed via QualtricsXM to practising optometrists in the UK via email lists and newsletters of local optical committees, social media, and optometric networks. Questions focussed on learning styles, training needs and barriers to learning.

**Results:**

Fifty-five respondents completed the survey. Forty-eight respondents answered the question on where they get information about myopia control and learning style, 79.2% indicated that conferences offering *Continuing Professional Development* (CPD) material were their main source, and 20.8% preferred online learning as the preferred format of delivery. Optometrists would like to receive training in clinical assessments (78.9%), evaluating suitable interventions (76.3%), developing and implementing specific patient intervention plans (76.3%), carrying out chosen myopia control interventions (fitting/prescribing) (73.7%), and the use of pharmacological interventions (94.4%). Of the 40 respondents who answered professional development questions, 97 5% were most interested in finding, identifying and applying evidencebased practice (EBP), followed by clinical decision-making in myopia control (95.0%). When asked about barriers to learning in this field, 29.7% reported limited time to attend training as the greatest barrier.

**Conclusion:**

Optometrists felt they need training in various aspects of myopia management, from practical skills to assessing and fitting/prescribing appropriate myopia control interventions. They were also interested in learning more on EBP and clinical decision-making related to myopia control. To improve the uptake of myopia control among optometrists, various learning methods, especially online learning, and providing sufficient time for training are crucial.

## Introduction

Myopia is considered an epidemic in some developed East Asian countries (Morgan et al. 2018), with prevalence reaching nearly 94% to 97% in some communities in China and South Korea in young adults aged 18–19 years (Jung et al. 2012; Zhang et al. 2022). It is predicted that, by 2050, the global prevalence will reach 50% (Holden et al. 2016). Wong and Dahlmann-Noor (2020) conducted a 10-year review from 2008 to 2017 of spectacle prescriptions for myopia in individuals under 17 years of age who had attended a secondary and tertiary eye care facility located in London, UK. Their findings showed that both the proportion of spectacle prescriptions for myopia and the rate of progression were higher than what had been previously documented for European countries (Wong & Dahlmann-Noor 2020). In another study conducted in Northern Ireland, UK, the prevalence and proportion of myopia in the white population was found to be relatively low (McCullough et al. 2016) compared to other worldwide studies (French et al. 2013). However, despite the low proportion, the number of myopic Caucasian children aged 10–16 years in the UK has increased more than twofold in the last 50 years (McCullough et al. 2016).

As the prevalence of myopia increases, the prevalence of pathology associated with myopia, such as cataract, glaucoma, retinal detachment and myopic macular degeneration, are expected to increase the incidence of visual impairment and irreversible blindness (Holden et al. 2016; Sankaridurg et al. 2021; Williams & Hammond 2019) across all severities of myopia (Flitcroft 2012; Mitchell et al. 1999; Younan et al. 2002). Associated medical, social and financial well-being damage, which has been shown to lower quality of life in myopic individuals, (Rose et al. 2000; Yokoi et al. 2014), will also likely increase in prevalence/severity. Given this, developing effective strategies for myopia management is becoming increasingly important (Bullimore & Brennan 2023).

At present, multiple interventions, encompassing optical, pharmacological and behavioural approaches, have been devised in an attempt to slow myopia progression amongst children (Bullimore & Richdale 2020) with some of these interventions now available in the UK market. Whilst evidence for their efficacy exists (Bullimore & Richdale 2020) their adoption remains low (Wolffsohn et al. 2020), with many optometrists still opting mainly for conventional lenses as their first line of myopia treatment (Wolffsohn et al. 2020). Though data would suggest that the adoption of myopia control strategies is rising, this is slow and there are substantial disparities between and within continents (Wolffsohn et al. 2023).

Previous studies and surveys have explored attitudes and practices related to the management of myopia in different countries (Douglass et al. 2020; Martínez-Pérez et al. 2023; McCrann et al. 2020; Nti et al. 2022), all of which provide valuable insights. However, there is a scarcity of published information on optometrists practising in the UK. Data collected from eye care practitioners (ECPs) in Ghana and Nigeria indicated a tendency to prescribe single vision lenses despite being aware of the various options available for myopia control (Nti et al. 2022). The findings of McCrann et al. (2020), who conducted focus group discussions with optometrist educators, optometry students and clinical optometrists in Ireland, revealed that these groups had not yet fully integrated myopia control into their clinical practice. Similar findings were also observed among Spanish optometrists, who only engage with myopia control interventions to a small extent (Martínez-Pérez et al. 2023). These findings suggest that optometrists may not be prescribing myopia control strategies as frequently as they could.

It is important to consider the differences in legislation, education, practice areas and healthcare delivery systems in the UK that could influence optometrists’ engagement in myopia control in the UK setting. Therefore, the data from other countries may not be directly applicable to the UK. Furthermore, the reasons for optometrists’ apparent resistance to myopia control in clinical practice are still largely unknown. One possible contributing factor could be the lack of training in myopia control. Therefore, it is important to explore the training needs that influence optometrists’ engagement in myopia control within this particular context.

In myopia management, optometrists often need to apply evidence-based practice (EBP). Evidence-based practice involves drawing upon current, reliable research evidence combined with patient preferences or circumstances, clinical environment factors and practitioner expertise as sources for practice strategies (Alnahedh et al. 2015; Hoffmann 2017). Alnahedh et al. (2015), reported that optometrists from Australia and Saudi Arabia require additional EBP training. However, there is limited information available on UK optometrists’ needs in terms of EBP knowledge and skills related to myopia management. Therefore, further investigation is required to explore whether UK optometrists have an interest in receiving training related to EBP for myopia management.

The aim of this study was to explore the skills and competencies perceived as required by optometrists in the UK to effectively facilitate their engagement in myopia control. Myopia control is now becoming the routine standard of care for children who show signs of myopia in large parts of the world. The results of this study will be of benefit to all eye care practitioners working with children and young adults by raising awareness of myopia control and training needs, before myopia control becomes mainstream in the UK as the number of myopic patients increases and more myopia control interventions become available.

## Materials and Methods

This study was reported based on the Checklist for Reporting Results of Internet E-Survey (CHERRIES) guidelines (Eysenbach 2004) and was approved by The University of Manchester Proportionate Research Ethics Committee (Ref: 2022-13187-22281). Procedures adhered to the tenet of the Declaration of Helsinki. The survey was a self-administered online open survey. The questionnaire could be accessed by participants through a direct link. There was a brief explanation regarding the study at the beginning of the survey, including a link to the full participant information sheet. Participation was entirely voluntary, and informed consent was obtained when participants started the survey. There were no incentives provided for participation.

### Survey Design and Development

A custom survey was developed to explore issues relating to optometrists’ learning needs and barriers to learning towards engaging with myopia control, specific to the UK myopia management landscape. The content and design of the questionnaire was informed by a review of other well-designed survey studies in similar optometry and healthcare fields (Douglass et al. 2020; Doyle et al. 2019; Schindel et al. 2019; Schmid et al. 2000). A list of themes and related questions was then compiled and modified into a preliminary instrument. Face and content validity were conducted using feedback from optometrists from the research group and a pilot test with seven randomly selected optometrists. Feedback from optometrists in the research group was sought for unclear and poorly worded questions, and appropriate changes were made until no further feedback was received. The responses from optometrists in the pilot test were not included in the results of the main study.

Cronbach’s alpha was calculated to ensure the reliability of the questionnaire. Cronbach’s alpha is the most widely used model for measuring the internal consistency of a test or scale (Cronbach 1951; Tavakol and Dennick 2011).The overall Cronbach’s alpha for the training needs items was as follows: Knowledge (0.92), Experience (0.90), Confidence (0.94) and Training Need (0.74). The Cronbach’s alpha for the other items on professional learning needs was 0.71. In general, reliability coefficients above 0.70 were considered reliable or acceptable, indicating that the items structured for optometrists’ training and professional learning needs were reliable. This was done prior to the distribution of the survey. However, a Rasch analysis was not conducted for this study.

The survey consisted of the following items to explore:

- Respondents’ demographics, including years practising as optometrists, time spent in practice, university from which they graduated with a degree in optometry, additional qualifications if they have them, type of primary workplace, and the city or area in which their primary practice is located. This section contained a combination of multiple-choice questions with an open text box as one of the choices, and short answer questions.
- Information attainment, consisting of questions asking respondents to select their sources of information on myopia control and their preferred method of learning from a multiple-choice question with an open text box as an ‘other’ choice option, if the respondents wished to add their own answers in addition to the given choices.
- Training needs analysis, consisting of 12 items on skills and competencies related to myopia control interventions. Respondents were asked to rate their knowledge, experience and confidence in relation to 12 items on a scale of 1 (low) to 5 (high). They were also asked to answer Yes/No if they needed training on the skills and competencies listed. An example scenario from (Schindel et al. 2019) was included to guide respondents in answering this section.
- Further professional learning needs, consisting of 7 items on further areas in which optometrists would be interested to learn more. Respondents were asked to choose, on a 5-point Likert scale, between 1 (not interested at all), 2 (somewhat not interested), 3 (neither interested nor not interested), 4 (somewhat interested) and 5 (very interested).
- Barriers to learning where respondents were asked to select answers from a multiple-choice question.

The questionnaire can be found in the supplementary file.

This study was part of a survey on ‘Optometrists’ knowledge, attitudes, readiness and learning needs towards engaging with myopia control’. The aim of this study was to explore the skills and competencies needed by UK optometrists to facilitate their engagement in myopia control intervention, their preferred learning styles and domains, and barriers to learning. The aims and hypotheses of this study differed from those of the attitudes study and there was no overlap in themes and outcomes.

The survey was hosted using the QualtricsXM web-based survey tool (Qualtrics, Provo, Utah). The survey was set up to be anonymous, and all data collected were treated as strictly confidential.

### Participant Recruitment

According to the General Optical Council (GOC), there are 16,670 registered optometrists in the UK in 2020 (GOC 2021). The sample size (n) was determined using the Raosoft software (Raosoft, Inc., Seattle, Washington) using the formula: n = NX / (X + N – 1), where, X = Z_α/2_^2^p(1–p) / MOE^2^; N = population size, Z_α/2_ = critical value of the Normal distribution at α/2, p = sample proportion and MOE = margin of error. The population size (N) was 16,670 with a critical value (Z_α/2_) of 1.48, for a confidence interval of 86. The sample proportion was 50%, giving the largest sample size with a margin of error of 10% to be 55.

It was difficult to know how many optometrists received the link to the survey as it was distributed to practising optometrists in the UK through various channels. These included various Local Optical Committees (LOC), who agreed to send the survey link information to their member lists and newsletters, social media, instant messaging platforms (Whatsapp) and other optometric networks with a link to the online survey in QualtricsXM.

Participants could exit the survey at any time and responses were automatically saved. They could continue answering the survey where they left it. However, the survey was set up so that participants could not go back and change their previous answers or give multiple submissions.

### Statistical Analysis

Data were downloaded into Microsoft Excel (Microsoft Corporation, Redmond, Washington), and analysis was carried out using Microsoft Excel and StatsiQ in QualtricsXM. Missing data and responses with less than 50% completion were not included in the analysis (Ball 2019). Analysis was completed on a question-by-question basis and included full and partially (>50%) completed responses. Descriptive analysis and chi-squared tests were used to describe frequencies and associations between optometrists’ characteristics and their responses to the areas of training needed. The threshold for statistical significance was (*P* < 0.05).

## Results

Fifty-eight participants responded to the survey. However, three participants who did not meet the threshold for inclusion in the study (>50% completeness) were excluded, leaving 55 participants who met the threshold for analysis. Analyses were conducted by comparing full completers with partial completers. No significant differences were found between the full and partial completers except for Items 8 and 9 for Knowledge and Confidence in the training needs analysis. Therefore, it was adequate to include partial completers in the analysis of this study. However, the number of respondents to each question varied, as shown in Table 1. A sample size of 55 participants provides 86% confidence interval and a 10% margin of error. The least number of responses for any question was 36 (item 12 on pharmacological myopia control) and this sample size gives a 77% confidence interval and a 10% margin of error. Apart from this one question, all other items had a confidence interval of around 80%.

**Table 1:** Table showing the themes of the questions, the question number and the number of respondents who answered the question.


THEMES		NUMBER OF RESPONDENTS (n)

Information attainment on myopia control		48

Preferred learning style		48

Self-rated Training Needs		

Rating on Knowledge	Item 1 to 7	40

	Item 8 to 12	39

Rating on Experience	Item 1 to 6	39

	Item 7 to 11	38

	Item 12	37

Rating on Confidence	Item 1 to 6	40

	Item 7 to 11	39

	Item 12	38

Training Needed? (Yes/No)	Item 1 to 6	38

	Item 7 to 11	37

	Item 12	36

Interests in further professional development	Item 1 to 6	40

	Item 7	39

Barriers to learning		37


Fifty-five respondents answered the question on how many years they have been practising as an optometrists. Mean years was 17.44 years (SD ± 11.87, range 1–42 years). More than half of the respondents work full time (56.4%, 31/55) and 38.2% (21/55) work part time. Those who answered ‘other’ (5.5%, 3/55) indicated that they work ad hoc or as locum.

Almost half of the respondents work in independent optometry practices (45.5%, 25/55), 34.5% (19/55) work in multiple (chain-owned) practices, 7.3% (4/55) work in academia, 5.5% (3/55) work in the hospital eye service and 7.3% (4/55) selected ‘other’. Free-text responses to the ‘other’ category included those working in more than one practice, in domiciliary practice, and in University Eye clinic. See Table 2 for further detail of respondent characteristics.

**Table 2:** Respondent characteristics including the years of experience, practice patterns and education.


CHARACTERISTICS	NUMBER OF RESPONDENTS (n)	PERCENTAGE (%)

**Years in practice (n = 54)** ^a^		

5–10	20	37.0

11–20	12	22.2

>20	22	40.7

		

**Time Spent in Practice (n = 55)**		

Full time	31	56.4

Part-time	21	38.2

Other Ad hoc/locum	3	5.5

**Undergraduate Optometry degree—University studied (n = 55)**		

Aston University	7	12.7

University of Bradford	11	20.0

Cardiff University	3	5.5

City University	1	1.8

Glasgow Caledonian University	12	21.8

The University of Manchester	14	25.5

Ulster University	3	5.5

Other Technological University Dublin/DIT Kevin Street, Dublin (n = 2) QUT Brisbane (n = 1) Hogeschool Utrecht (n = 1)	4	7.3

		

**Additional Qualifications (n = 27)** ^b^		

Professional Certificate	11	40.7

Professional Higher Certificate	4	14.8

Postgraduate Certificate	3	11.1

Independent Prescribing	13	48.1

MSc	7	25.9

PhD	3	11.1

Other WOPEC MECS (n = 1) Nesgat glaucoma qualification (n = 1) FBDO CL (n = 1) School Vision diploma, sports vision diploma and various WOPEC qualifications (n = 1) Working towards IP qualification (n = 1)	5	18.5

More than 1 additional qualifications	12	44.4

**Primary workplace (n = 55)**		

Academia	4	7.3

Hospital	3	5.5

Independent	25	45.5

Multiple	19	34.5

Other Hospital, independent & research in atropine myopia (n = 1) Domiciliary (n = 1) Independent and hospital (n = 1) University eye clinic (n = 1)	4	7.3

**Location of primary practice (n = 55)**		

North East and Yorkshire	24	43.6

North West	9	16.4

Midlands	4	7.3

East of England	1	1.8

London	1	1.8

South East	2	3.6

South West	2	3.6

Scotland	12	21.8


^a^ One out of the 55 respondents did not respond to this question. ^b^ Participants were allowed to choose >1 option. DIT = Dublin Institute of Technology; QUT = Queensland University of Technology; WOPEC = Wales Optometry Postgraduate Education Centre; MECS = Minor Eye Conditions Service; FBDO CL = Fellowship Diploma in Ophthalmic Dispensing; IP = Independent Prescribing.

### Information attainment and preferred method of learning

**Figure 1:**
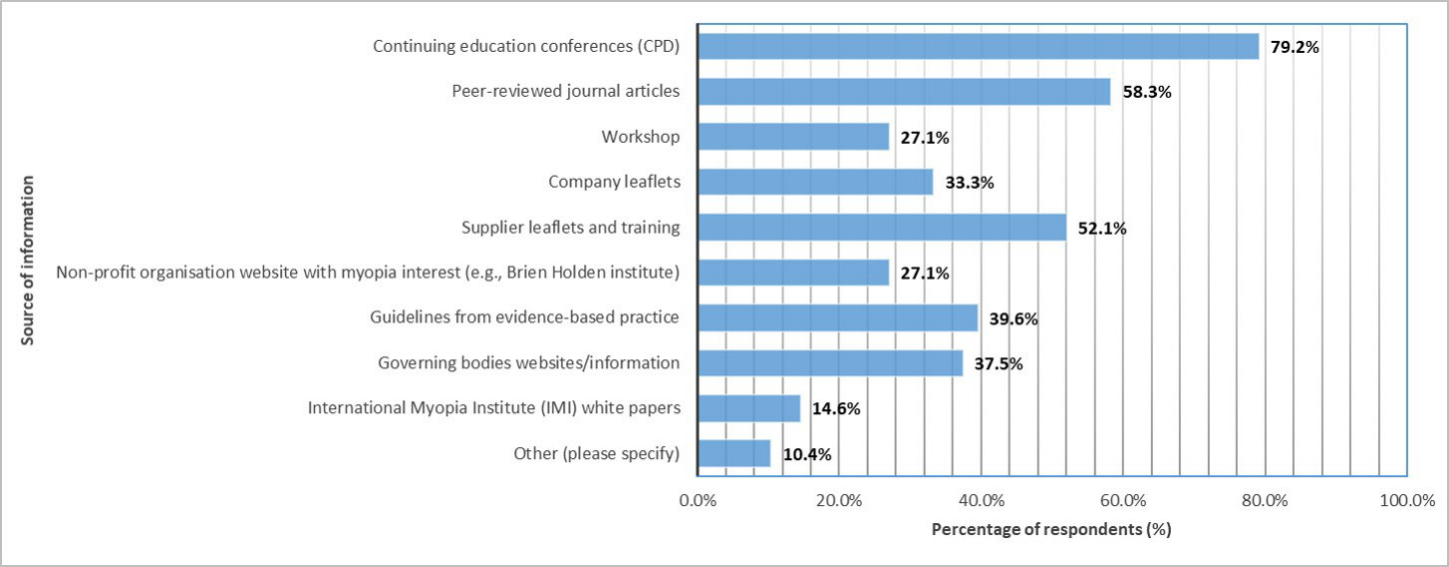
Preferred source of information or training as a function of percentage of respondents (n = 48).

Forty-eight respondents answered the question about where they receive their knowledge regarding myopia control. Respondents were able to make multiple selections. The preferred source of information was through continuous education conferences offering *Continuing Professional Development* (CPD) material (79.2%, 38/48), followed by peer-reviewed journal articles (58.3%, 28/48). The least chosen source of information was from the International Myopia Institute (IMI) white papers (14.6%, 7/48) and other sources (10.4%, 5/48). Those who chose other sources indicated their source of information was from colleagues and optometry social media groups (see Figure 1).

**Figure 2:**
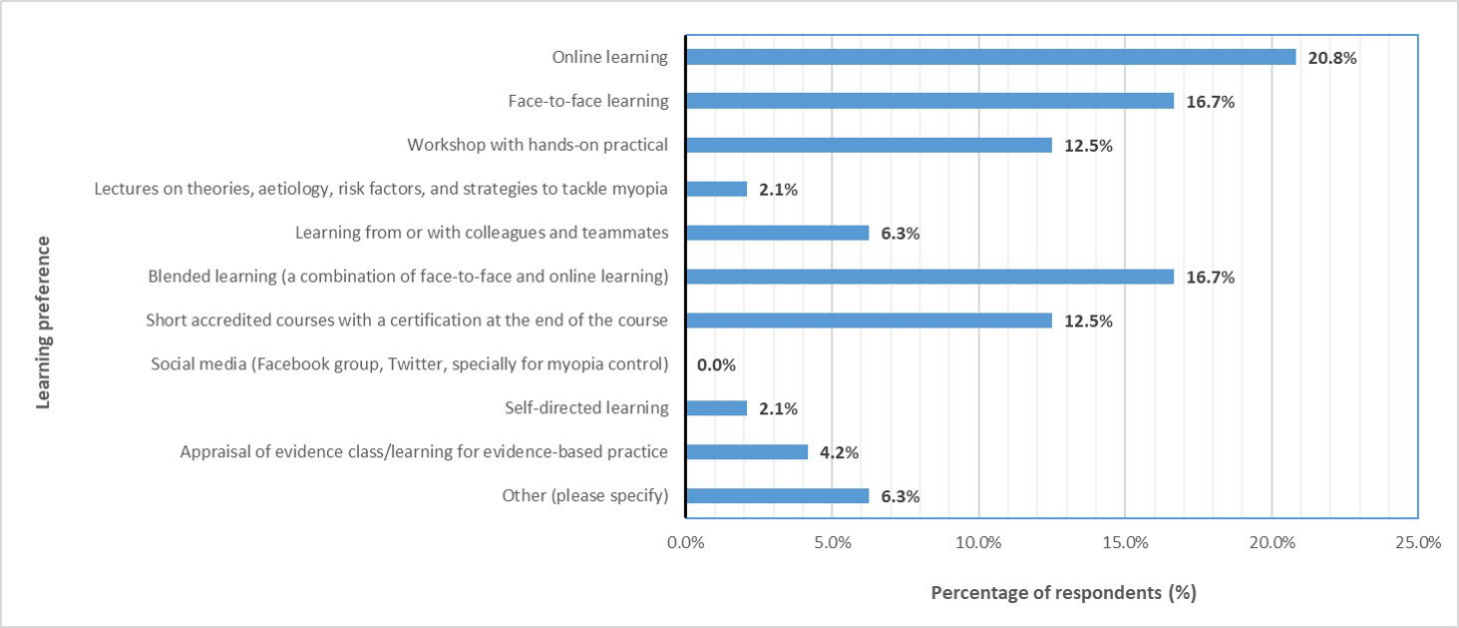
Preferred type of learning delivery as a function of percentage of respondents (n = 48).

Forty-eight respondents answered the question related to the preferred method of learning. Online learning scored highest (20.8%, 10/48) as their most preferred learning format, followed by face-to-face learning and blended learning (a combination of face-to-face and online learning) both with 16.7% (8/48). Workshops with hands-on practical and short accredited courses with certificates shared the same number of respondents who chose those methods as their preferred learning style (12.5%, 6/48). Some respondents (6.3%, 3/48) preferred to learn from or with their colleagues and teammates. A small proportion of respondents chose to learn via appraisal of evidence class (4.2%, 2/48), lectures (2.1%, 1/48), and self-directed learning (2.1%, 1/48). None had chosen social media such as Facebook groups and Twitter specifically for myopia control as their preferred way of learning (see Figure 2). Responses under ‘other’ included journal articles, preferred all options given, and one respondent (due to a technical error) indicated that the question was invalid as no more than one option could be selected (see Figure 2).

### Self-reported knowledge, experience and confidence in skills related to myopia control and training needed

In this section, respondents were asked to self-rate their knowledge (K), experience (E) and confidence (C) on a scale of 1 (low) to 5 (high), with 3 as average, and to indicate whether they needed training (Yes/No) on a particular skill related to myopia management/control. Analyses between full and partial completers revealed significant differences in Items 8 and 9 for Knowledge between full and partial completers (Mann-Whitney, *P* = 0.039; *P* = 0.031). Significant differences between full and partial completers were also found in items 8 and 9 for Confidence (Mann-Whitney, *P* = 0.019; *P* = 0.004).

The number of respondents who answered this section varied (see Table 1). The number of respondents who rated their knowledge (K), experience (E) and confidence (C) in evaluating, developing, implementing and carrying out myopia control interventions as high (4 and 5) was mostly around 30-40% for each skill/competency. This was except for knowledge in evaluating patient suitability for myopia control and knowledge in carrying out the chosen myopia control intervention, where a greater number of respondents rated their knowledge in these two skill areas as high (see Table 3).

**Table 3:** The proportion of respondents who reported knowledge, experience and confidence as high (score of 4 or more) for specific skills/competencies.


ITEM	SKILLS/COMPETENCIES	KNOWLEDGE n^a^ (%)	EXPERIENCE n^a^ (%)	CONFIDENCE n^a^ (%)	TRAINING NEEDED n^a^ (% YES)

1	Collect relevant patient information (e.g., history taking, including parental myopia, behavioural factors such as time spent outdoors, time spent near work, etc.)	39 (97.5)	31 (79.5)	35 (87.5)	7 (18.4)

2	Evaluate the suitability of myopia control interventions to be implemented	22 (55.0)	13 (33.3)	15 (37.5)	29 (76.3)

3	Perform required assessment for myopia control intervention	14 (35.0)	9 (23.1)	12 (30.0)	30 (78.9)

4	Develop specific patient intervention plans	16 (40.0)	13 (33.3)	15 (37.5)	29 (76.3)

5	Implement specific patient intervention plans	15 (37.5)	13 (33.3)	15 (37.5)	29 (76.3)

6	Carry out chosen myopia control intervention	19 (47.5)	14 (35.9)	16 (40.0)	28 (73.7)

7	Monitor and follow up intervention plan	18 (45.0)	14 (36.8)	13 (33.3)	28 (75.7)

8	Proper patient documentation for the medico-legal aspect	14 (35.9)	12 (31.6)	12 (30.8)	28 (75.7)

9	Change of intervention plan when the initial plan does not work out	10 (25.6)	7 (18.4)	9 (23.1)	32 (86.5)

10	Co-management with other health care providers	13 (33.3)	9 (23.7)	11 (28.2)	28 (75.7)

11	Provide parents, caregivers, and patients with information regarding their interventions	26 (66.7)	21 (55.3)	23 (59.0)	22 (59.5)

12	Usage of pharmacological intervention (e.g., low dose atropine)	9 (23.1)	4 (10.8)	5 (13.2)	34 (94.4)


^a^ n = 36–40 due to missing data.

More than half of the respondents rated their knowledge (K = 55.0%, 22/40) as high when evaluating patients as suitable or unsuitable for myopia control, but less than half did so for experience and confidence (E = 33.3%, 13/39; C = 37.5%, 15/40). When carrying out the chosen myopia control intervention, a greater number of respondents rated their knowledge and confidence as high (K = 47.5%, 19/40; C = 40.0%, 16/40), while experience was lower (E = 35.9%, 14/39). When developing and implementing specific intervention plans for patients, less than half of the respondents rated their knowledge, experience and confidence as high (Develop: K = 40.0%, 16/40; E = 33.3%, 13/39; C = 37.5%, 15/40), (Implement: K = 37.5%, 15/40; E = 33.3%, 13/39; C = 37.5%, 15/40).

A number of respondents rated their knowledge, experience and confidence (K = 35.0%, 14/40; E = 23.1%, 9/39; C = 30.0%, 12/40) in practical skills as high in the assessment related to myopia control intervention and included the example of additional tools (if available) in their practice, e.g. corneal topography, Lenstar and IOL Master and in co-management with other healthcare providers (K = 33.3%, 13/39; E = 23.7%, 9/38; C = 28.2%, 11/39).

When it came to collecting relevant patient information (e.g., history taking, including parental myopia, behavioural factors such as time spent outdoors, time spent doing near work, etc.), almost all respondents rated their knowledge as high (K = 97.5%, 39/40) and a greater number of respondents also felt experienced and confident in this area of competence (E = 79.5%, 31/39; C = 87.5%, 35/40). More than half of the respondents rated their knowledge, experience and confidence as high when it came to providing parents, carers and patients with information about their interventions (K = 66.7%, 26/39; E = 55.3%, 21/38; C = 59.0%, 23/39) (see Table 3).

The proportion of respondents who rated their knowledge, experience and confidence as high was lower for the ability to change the intervention plan if the initial plan did not work for the patients (K = 25.6%, 10/39; E = 18.4%, 7/38; C = 23.1%, 9/39). The same was true for the skills they perceived in using pharmacological interventions (e.g., low dose atropine) as they were not allowed to prescribe atropine for myopia control (K = 23.1%, 9/39; E = 10.8%, 4/37; C = 13.2%, 5/38) (see Table 3).

In terms of training needs, all respondents, regardless of their years of practice, time spent in practice and their primary workplace, indicated that they need training in all areas/skills related to myopia management, as shown in Table 4. There was a significant association between years of experience as a practising optometrist and the use of pharmacological interventions (*P* = 0.047). There was also a significant association between those working in independent and multiple practice with skills in carrying out chosen MC interventions (*P* = 0.042) (Table 4).

**Table 4:** Specific skills for which optometrists answered ‘Yes’ when asked if they needed training in the field, by number of years in practice, time spent in practice and primary practice type.


	EVALUATE THE SUITABILITY OF MC INTERVENTIONS TO BE IMPLEMENTED	PERFORM REQUIRED ASSESSMENT FOR MC INTERVENTION	DEVELOP SPECIFIC PATIENT INTERVENTION PLANS	IMPLEMENT SPECIFIC PATIENT INTERVENTION PLAN	CARRY OUT CHOSEN MC INTERVENTION	MONITOR AND FOLLOW UP INTERVENTION PLAN	PROPER PATIENT DOCUMENTATION FOR THE MEDICO-LEGAL ASPECT	CHANGE OF INTERVENTION PLAN WHEN THE INITIAL PLAN DOES NOT WORK OUT	CO-MANAGEMENT WITH OTHER HEALTH CARE PROVIDERS	PROVIDE PARENTS, CAREGIVERS, AND PATIENTS WITH INFORMATION REGARDING THEIR INTERVENTIONS	USAGE OF PHARMACOLOGICAL INTERVENTION

**Years in practice**	n = 37 (Yes, n = 28)	n = 37 (Yes, n = 29)	n = 37 (Yes, n = 28)	n = 37 (Yes, n = 28)	n = 37 (Yes, n = 27)	n = 36 (Yes, n = 27)	n = 36 (Yes, n = 27)	n = 36 (Yes, n = 31)	n = 36 (Yes, n = 27)		n = 35 (Yes, n = 33)

5–10	11 (39.3)	11 (37.9)	11 (39.3)	11 (39.3)	11 (40.7)	10 (37.0)	10 (37.0)	11 (35.5)	11 (40.7)		11 (33.3)

11—20	6 (21.4)	7 (24.1)	7 (25.0)	7 (25.0)	6 (22.2)	7 (25.9)	7 (25.9)	8 (25.8)	6 (22.2)		7 (21.2)

>20	11 (39.3)	11 (37.9)	10 (35.7)	10 (35.7)	10 (37.0)	10 (37.0)	10 (37.0)	12 (38.7)	10 (37.0)		15 (45.5)

*P-value^a^*	0.218	0.389	0.286	0.286	0.194	0.337	0.337	0.279	0.066		**0.047**

**Time Spent in Practice**	n = 36 (Yes, n = 27)	n = 36 (Yes, n = 28)	n = 36 (Yes, n = 27)	n = 36 (Yes, n = 27)	n = 36 (Yes, n = 26)	n = 35 (Yes, n = 26)	n = 35 (Yes, n = 26)	n = 35 (Yes, n = 30)	n = 35 (Yes, n = 26)	n = 35 (Yes, n = 21)	n = 34 (Yes, n = 32)

Full time	14 (51.9)	14 (50.0)	13 (48.1)	14 (51.9)	13 (50.0)	12 (46.2)	15 (57.7)	15 (50.0)	13 (50.0)	11 (52.4)	17 (53.1)

Part-time	13 (48.1)	14 (50.0)	14 (51.9)	13 (48.1)	13 (50.0)	14 (53.8)	11 (42.3)	15 (50.0)	13 (50.0)	10 (47.6)	15 (46.9)

*P-value^b^*	1.000	1.000	1.000	1.000	1.000	0.711	0.121	1.000	1.000	0.733	0.485

**Primary workplace**	n = 30 (Yes, n = 22)	n = 30 (Yes, n = 23)	n = 30 (Yes, n = 22)	n = 30 (Yes, n = 22)	n = 30 (Yes, n = 21)	n = 29 (Yes, n = 21)	n = 29 (Yes, n = 22)	n = 29 (Yes, n = 25)	n = 29 (Yes, n = 21)	n = 29 (Yes, n = 17)	n = 28 (Yes, n = 27)

Independent	11 (50.0)	12 (52.2)	10 (45.5)	10 (45.5)	9 (42.9)	11 (52.4)	11 (50.0)	13 (52.0)	10 (47.6)	7 (41.2)	16 (59.3)

Multiple	11 (50.0)	11 (47.8)	12 (54.5)	12 (54.5)	12 (57.1)	10 (47.6)	11 (50.0)	12 (48.0)	11 (52.4)	10 (58.8)	11 (40.7)

*P-value^b^*	0.407	0.427	0.092	0.092	**0.042**	0.408	0.187	0.121	0.093	0.053	1.000


^a^Chi-square test; ^b^Fisher’s exact test; Analyses were only conducted for items with ≥10 responses. MC = myopia control.

### Further professional development and barriers to learning

In this section, respondents were asked to rate how interested they are in further professional development in the following categories shown in Table 5. They rated their interests in specific further professional development using a 5-point Likert scale from 1 (not interested at all) to 5 (very interested). Forty respondents answered items 1 to 6, while 39 answered item 7 (see Table 1). The highest proportion of respondents reported their interest in finding, identifying, and applying the best EBP on myopia control (97.5%, 39/40), followed by clinical decision-making on myopia control (95%, 38/40) (Table 5). Conversely, respondents were least interested in furthering complementary and alternative medicines (50%, 20/40). Complementary and alternative medicine here refers to acupuncture (Wei et al. 2011) and nutritional supplements (Trier et al. 2008) for slowing the progression of myopia.

**Table 5:** The proportion of respondents who reported their interests in specific further professional development (score 4 and above).


ITEM	AREA FOR FURTHER PROFESSIONAL DEVELOPMENT	n^a^ (%)

1	Therapeutics clinical knowledge	34 (85.0)

2	Complementary and alternative medicines	20 (50.0)

3	Clinical decision-making on myopia control	38 (95.0)

4	Communication and negotiation skills, especially in communicating with myopic patients and/or parents/guardian	35 (87.5)

5	Coaching for giving support and motivation to patients or parents to increase compliance to interventions	33 (82.5)

6	Finding, identifying, and applying best EBP on myopia control	39 (97.5)

7	Teamwork skills for co-managing myopic patients	36 (92.3)


^a^ n = 39–40 due to missing data.

**Figure 3:**
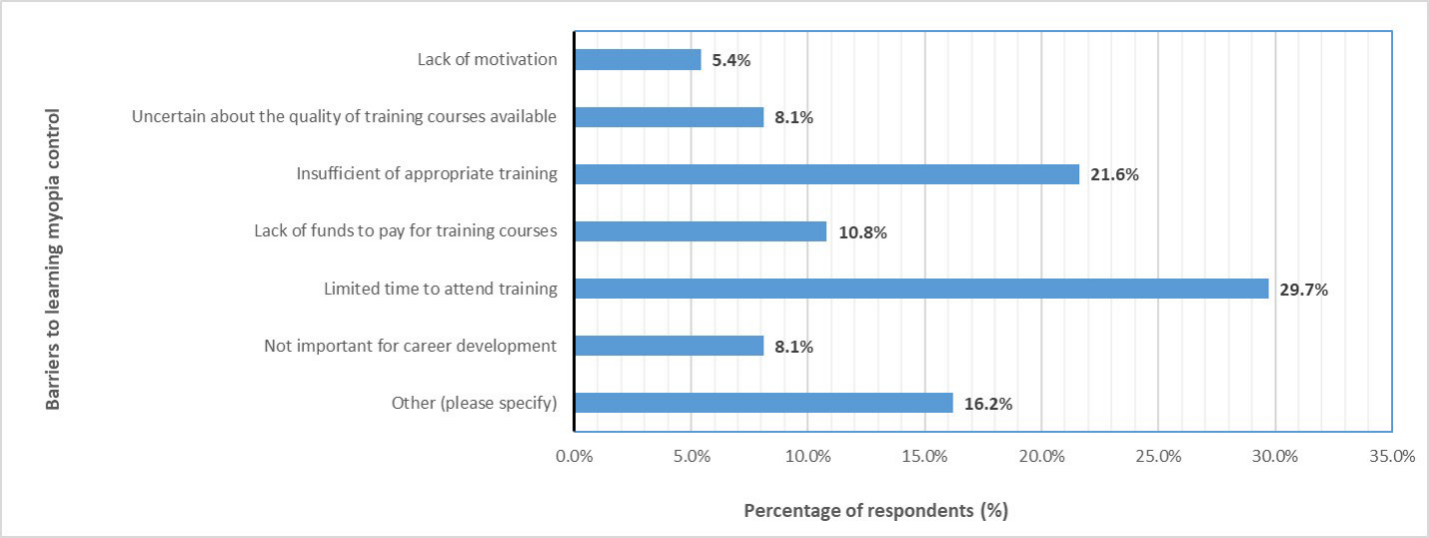
Barriers to learning myopia control as a function of percentage of respondents (n = 37).

Thirty-seven respondents answered the question about barriers to learning myopia control. Respondents could select one option from the list of options given. Almost 30% of respondents indicated that it was due to limited time to attend training (n = 11), followed by insufficient availability of appropriate training (21.6%, 8/37). Due to technical error, the free text box for the option ‘other’ was not available and, therefore, responses from six respondents who selected ‘other’ could not be examined in more detail. The remaining respondents indicated that they lacked financial resources to attend training courses (10.8%, 4/37), that they were uncertain about the quality of training offered, and that they did not consider learning myopia control to be important for their professional development (each 8.1%, 3/37). The least selected barrier to learning myopia control was lack of motivation to learn (5.4%, 2/37) (see Figure 3).

## Discussion

The majority of optometrists in this study rated themselves as knowledgeable, experienced and confident in history taking and consultation with myopic patients and parents or carers. However, their confidence in technical skills and competencies related to myopia control, like evaluating interventions and implementing tailored plans, and fitting or prescribing myopia control interventions, was low, with less than 50% of the participants feeling confident. This might be due to the fact that myopia control interventions are still a new area for optometrists (Bullimore & Richdale 2020; McCrann et al. 2020), and they have not yet gained sufficient exposure to clinical work in this area. It is also novel for many optometrists, with many graduating from University prior to the establishment of myopia management interventions. In addition, this area of practice is changing all the time as new interventions (Bao et al. 2022) and updated research for myopia control (Chamberlain et al. 2022) become available, adding further challenge to optometrists as they need to update their knowledge and skills continuously (Wildsoet et al. 2019). This is the case even for those not actively prescribing myopia control, with information from the College of Optometrists guidelines stating that optometrists should be able to discuss options and treatment with patients or parents and carers (College of Optometrists 2022).

Whilst most optometrists felt knowledgeable, they lacked experience and confidence in skills like assessing patients’ suitability for myopia control. This likely stems from limited exposure to young myopic patients. To cater to this need, learning through simulation could be suggested. Simulation is a great learning method where learners can apply their knowledge and skills in a controlled, safe environment, and it is widely used in medical education (Choong & Tan 2019; Sadideen et al. 2014). In optometry, simulated learning experience is already being used, for example, in using virtual refractor (Alhazmi et al. 2018; Woodman-Pieterse et al. 2016). Ten optometry students who used the virtual refractor reported feeling more knowledgeable and confident, achieving refractive accuracy of within 0.22 ± 0.22 DS and 13% faster than their peers in the control group. It is a great learning experience to simulate cases of myopia where optometrists would need to apply myopia management. This could probably give optometrists experience and skills in managing myopia more appropriately and increase their confidence.

Optometrists in this study also rated their confidence in communicating with patients, parents or carers during history taking as high. Communication is one of the key skills for healthcare professions (Shah et al. 2021) and a core competency in optometry practice (GOC 2020), so confidence in this area is perhaps unsurprising.

The results suggest that optometrists are keen to be trained in the usage of low-dose atropine for myopia control. There is now positive UK trial data on the efficacy of low-dose atropine (0.01%) (Zadnik et al. 2023). However, this does not necessarily mean that optometrists in the UK will be able to prescribe drugs specifically for myopia control in the near future. As our sample size was small, the findings cannot be generalised to the optometry profession as a whole. Our findings tally with a study of Singaporean ECPs who preferred atropine as a means of myopia control (Yang et al. 2022). However, atropine can only be prescribed by ophthalmologists, and the actual most dispensed myopia control intervention in Yang et al. was myopia control spectacles.

The preferred source of information regarding myopia control was from attendance at continuing education conferences. This is consistent with the findings in Australia, where more than 80 percent of optometrists considered continuing education conferences and events to be very important information sources in their clinical practices regarding childhood myopia (Douglass et al. 2020); it is also consistent with a study on age-related macular degeneration, where 62.9% ECPs in the UK obtained their sources of information from conference presentations (Lawrenson & Evans 2013). From continuing education conferences, attendees usually get the latest information, product knowledge and networking with peers from similar fields of interest (Kamal et al. 2022; Mishra 2016; Schreiber & Dole 2012), especially in the fast-growing field of myopia research (Martínez-Pérez et al. 2023; Wolffsohn et al. 2020). However, care must be taken as continuing education conferences can be influenced by the interests of sponsors/organisers (Mishra 2016). Optometrists who get their information from the industry sponsored events could be more biased towards the products in their clinical practice rather than adopting purely evidence-based practice (Downie et al. 2016). Furthermore, conferences may also be more business oriented rather than targeting the needs of training for specific professional skills (Barrett & Loughman 2018).

The present study showed around half of the respondents prefer guidelines as a source of information for myopia management, such as guidelines from EBP and governing bodies’ websites, and a smaller number of respondents choose IMI white papers. Myopia management guidelines have been published by the International Myopia Institute (IMI) (Jones et al. 2019) and the College of Optometrists (College of Optometrists 2022) and are freely available on their websites. However, from this survey, it appears that information from the IMI white papers is not being used widely. This may be due to the complexity and the way the guidelines were written, which some optometrists might find difficult to understand and vague to interpret. In addition, optometrists would need to use more EBP based on the guidelines provided by the College of Optometrists.

The preferred way of learning found in this study was online learning. Through online learning, learners can choose when they want to learn, the content they want to learn, the pace they want to learn, the amount of time they want to spend on it, and usually, even the type of media they want to use. In addition, online learning can be equally effective as instructor-led learning, like lectures in various medical education contexts (Ruiz et al. 2006) and optometry (Gupta and Gupta 2016). Other learning styles that the optometrists preferred were face-to-face and blended learning. A study on Singaporean ECPs showed they required more hands-on workshops on myopia (Yang et al. 2022), but their study included all ECPs, including ophthalmologists and opticians, and did not specifically measure optometrists’ preferences. A different study of Singaporean optometrists revealed that the preferred way of continuing professional education was through blended learning and eLearning (George et al. 2019). Several studies proved that interactive training using multiple methods is an effective method of delivering education in many other fields of medical education (Huang et al. 2019; Mansouri & Lockyer 2007). Findings from this current study also showed that there is variation in preferred learning style. This information can be used by the education and training providers for finding where they should be versatile in their approach and resources.

When asked about which specific further professional development areas the optometrists were interested in, nearly all participants (39 out of 40 respondents) were most interested in finding, identifying and applying EBP on myopia control. It is not surprising that this is the area that optometrists chose to further their professional development as research in myopia has increased rapidly in recent years, with various myopia control interventions being developed (Bullimore & Richdale 2020; Wildsoet et al. 2019). Thus, optometrists in this study were aware that they needed the skills to determine and apply the best practice evidence. This could be done by encouraging optometrists to engage with CPD with courses on EBP training and guidelines specifically for myopia control.

Regarding barriers to learning, our findings are consistent with other studies in which optometrists reported having limited time to attend training (Douglass et al. 2020; Needle et al. 2008). In a recent GOC registrant workforce survey, a high proportion of optometrists (40%) reported a desire to gain additional qualifications or skills, which showed they were more likely to attend or need training in the future (GOC 2022). It is important to note that the GOC workforce survey consisted primarily of optometry students and optometrists who work in hospitals as their majority of respondents; in this study, respondents are mostly from independent practice. Therefore, this current study may be more representative of the optometrists who deal more with young myopic patients although not generalisable to the whole optometric community.

Little is known on the allocated time or protected time for optometrists to attend training when they are working. Studies on the importance of lifelong learning and CPD for nurses have confirmed that CPD is important (Mlambo et al. 2021; Price & Reichert 2017), and a study on general practitioners in Ireland showed that CPD is perceived as beneficial to patient care by the majority of the surveyed GPs (McBride et al. 2022). However, the barriers to do this are the difficulties in getting time off or allocated time for study or attend CPD due to workload and low support from the senior management (Mlambo et al. 2021; Price & Reichert 2017).

To date, limited information has been published on the learning/training needs of optometrists in the UK in the niche of myopia control. The present study sought to explore this area. However, the limitations of this study are that the sample size was small and that it was a self-reported survey. Furthermore, due to the disruption caused by COVID-19, more research has been conducted in recent years involving surveys as their methods of data collection. This has led to called survey fatigue, where respondents refuse to answer, or response rates are low, due to too many surveys being answered (de Koning et al. 2021). Despite the small sample size of this study, it does highlight the need for additional research that explores what skills/competencies optometrists need, what learning methods they prefer, what areas they want to develop, and what barriers exist to learning myopia control interventions. A global survey on myopia management also yielded a quite similar number of respondents from the UK (n = 67) (Wolffsohn et al. 2023).

A further limitation is that the example given for the practical skills question (corneal topographer, Lenstar, IOL Master) may not be available in most optometrists’ practices. These instruments may be available in hospitals or academic institutions. Therefore, fewer optometrists in this cohort felt they had the knowledge, experience and confidence to use these instruments. However, employers or training providers may take this information into account when considering using these instruments in future training and having optometrists trained on these instruments.

The difference in the number of respondents to each question could be due to the setting of the questionnaire. Respondents could not return to the previous question if they had missed to answer it before moving on to the next question. This could be the reason for the difference in the number of responses to each question. In future studies, the survey could be set up in a way that respondents cannot move to the next question until they have answered the current question (forced answering), so that a more consistent response for each question could be expected.

In addition, future studies may include questions related to the specific optometry practice settings, such as hospital, independent and multiple. Questions could aim to understand how optometrists manage myopia or what myopia control strategies they recommend in different practice settings. Furthermore, it would be valuable to ask about the preferred method of myopia control among optometrists, as this information could guide the teaching focus for training providers and facilitate the availability of myopia control, making it easier to be offered in more practice settings.

## Conclusion

In conclusion, the optometrists who participated in this survey reported they receive information about myopia control interventions primarily at continuous education conferences that offered continuous professional development (CPD) points. They preferred online learning, and they needed training to improve their knowledge of myopia control in all areas of myopia management, from practical clinical skills to prescribing the chosen myopia control interventions. For further professional development, optometrists were particularly interested in EBP in myopia control. Training and education providers from academia, governing bodies and manufacturers can focus on these skills to provide optometrists with a balanced and preferred learning channel to improve optometrists’ uptake of myopia control interventions, although there is no clear single preferred method and diversity of learning style is key.

## Additional file

The additional file for this article can be found as follows:

10.22599/bioj.341.s1 Supplementary File. Optometrists’ Knowledge, Attitudes, Readiness and Learning Needs towards engaging with Myopia Control Questionnaire.
