# Supplementary material for: UK Optometrists’ Professional Learning Needs Toward Engaging with Myopia Control Interventions
Source: Br Ir Orthopt J. 2024 Feb 7;20(1):69–84. doi: 10.22599/bioj.341 (PMC10854455; doi:10.22599/bioj.341)
Supplement: Supplementary File. — Optometrists’ Knowledge, Attitudes, Readiness and Learning Needs towards engaging with Myopia Control Questionnaire. [file bioj-20-1-341-s1.pdf]

Participants Demographics

1) For how many years have you been a practicing Optometrist? __________

2) From the following list, please (√) the time you spend in practice:

- ☐ Full time
- ☐ Part-time
- ☐ Other (please specify): __________

3) From the following list, please (√) at which university did you undertake your undergraduate degree:

- ☐ Anglia Ruskin University
- ☐ Aston University
- ☐ University of Bradford
- ☐ Cardiff University
- ☐ City University
- ☐ Glasgow Caledonian University
- ☐ Huddersfield University
- ☐ Plymouth University
- ☐ University of Central Lancashire
- ☐ University of Manchester
- ☐ Teesside University
- ☐ University of Hertfordshire
- ☐ University of Highlands and Islands
- ☐ University of the West of England
- ☐ Ulster University
- ☐ Other (please specify): __________

4) From the following list, please (√) if you have additional qualifications related to Optometry. For example, Professional Diploma (Paediatric)

- ☐ MSc
- ☐ MPhil
- ☐ PhD
- ☐ Independent Prescribing
- ☐ Professional Certificate (________________)
- ☐ Professional Higher Certificate (_________________)
- ☐ Professional Diploma (_________________)
- ☐ Postgraduate Certificate (__________________)
- ☐ Other (please specify): __________

5) Primary workplace type of practice?

- ☐ Academia
- ☐ Research
- ☐ Hospital
- ☐ Independent
- ☐ Industry
- ☐ Multiple
- ☐ Other (please specify): __________

6) Please state the city or area in which your primary practice is located:

____

Information attainment and training needs analysis

1) From the following, choose from where do you get information regarding myopia control. You can select more than one option.

- ☐ Continuous education conference
- ☐ Peer-reviewed journal articles
- ☐ Workshop
- ☐ Company leaflets
- ☐ Supplier leaflets and training
- ☐ Non-profit organisation website with myopia interest (e.g., Brien Holden institute)
- ☐ Guidelines from evidence-based practice
- ☐ Governing bodies websites/information
- ☐ International Myopia Institute (IMI) white papers
- ☐ Other (please specify): __________

2) From the following, choose your preferred way of learning if you want to implement myopia control at your workplace. You can select more than one option.

- ☐ Online learning
- ☐ Face-to-face learning
- ☐ Workshop with hands-on practical
- ☐ Lectures on theories, aetiology, risk factors, and strategies to tackle myopia
- ☐ Learning from or with colleagues and teammates
- ☐ Blended learning (a combination of face-to-face and online learning)
- ☐ Short accredited courses with a certification at the end of the course
- ☐ Social media (Facebook group and Twitter, specially for myopia control)
- ☐ Self-directed learning
- ☐ Appraisal of evidence class/learning for evidence-based practice
- ☐ Other (please specify): __________

Training Needs Analysis

Items 1 - 12

Please indicate your current knowledge, experience, and confidence with the skills/competencies listed below, and whether you would like to receive additional training (e.g., lectures, practicals, workshop) in those skills/competencies.

Example, adapted from Schindel et al. 2019:

Select one option in each of the four columns below:

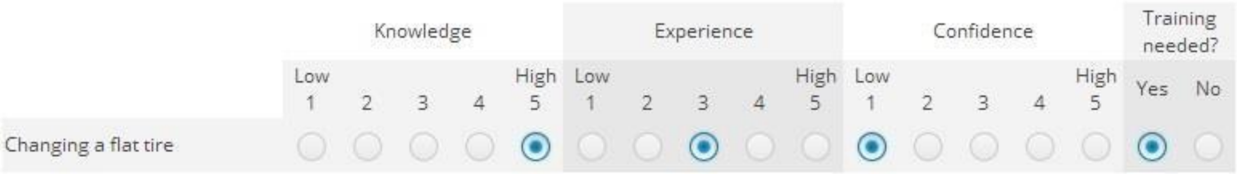

This means that today I have a great deal of knowledge (response of 5) about changing a flat tire, I have an average amount of experience (response of 3) with changing a flat tire, I am not confident (response of 1) in my ability to change a flat tire. It also means I would like additional training about changing a flat tire.

Now continue to select your ratings for each of the skills/competencies below.

No	Skills/competencies	Knowledge					Experience					Confidence					Training needed?	
		Low 1	2	3	4	High 5	Low 1	2	3	4	High 5	Low 1	2	3	4	High 5	Yes	No
1	Collect relevant patient information (e.g., history taking, including parental myopia, behavioural factors such as time spent outdoors, time spent near work, etc.)																	
2	Evaluate the suitability of myopia control interventions to be implemented																	
3	Perform required assessment for myopia control intervention (e.g., corneal topo, Lenstar, IOL Master)																	
4	Develop specific patient intervention plans																	
5	Implement specific patient intervention plans																	
6	Carry out chosen myopia control intervention																	
7	Monitor and follow up intervention plan																	

8	Proper patient documentation for the medico-legal aspect																	
9	Change of intervention plan when the initial plan does not work out																	
10	Co-management with other health care providers																	
11	Provide parents, caregivers, and patients with information regarding their interventions																	
12	Usage of pharmacological intervention (e.g., low dose atropine)																	

Further professional development

Items 1 - 7

Please indicate how interested you are in further professional development in each of the following categories. Please answer the following statement using the following scale:

5 = very interested 4 = somewhat interested, 3 = neither interested nor not interested, 2 = somewhat not interested, to **1 = not interested at all**

No	Statement	Scale				
1	Therapeutics clinical knowledge	5	4	3	2	1
2	Complementary and alternative medicines	5	4	3	2	1
3	Clinical decision making on myopia control	5	4	3	2	1
4	Communication and negotiation skills, especially in communicating with myopic patients and/or parents/guardian	5	4	3	2	1
5	Coaching for giving support and motivation to patients or parents to increase compliance to interventions.	5	4	3	2	1
6	Finding, identifying, and applying best evidence-based practice on myopia control.	5	4	3	2	1
7	Teamwork skills for co-managing myopic patients	5	4	3	2	1

Barriers to Learning

1) From the following, choose your current barriers to learning myopia control or specify other options not listed here. You can select more than one option.

- ☐ Lack of motivation
- ☐ Uncertain about the quality of training courses available
- ☐ Insufficient of appropriate training
- ☐ Lack of funds to pay for training courses
- ☐ Limited time to attend training
- ☐ Not important for career development
- ☐ Other (please specify): __________

We thank you for your time spent taking this survey.

Your response has been recorded.
